# Recommendations to facilitate the ideal fit note: are they achievable in practice?

**DOI:** 10.1186/s12875-015-0360-4

**Published:** 2015-10-13

**Authors:** Carol Coole, Fiona Nouri, Iskra Potgieter, Paul J. Watson, Louise Thomson, Rob Hampton, Avril Drummond

**Affiliations:** School of Health Sciences, University of Nottingham,, A Floor, Medical School, Queens Medical Centre, Nottingham, NG7 2HA UK; Research Design Service for East Midlands, School of Medicine, C Floor, Room 2400, Queen’s Medical Centre, Nottingham, NG7 2UH UK; New Academic Unit, Leicester General Hospital, Gwendolen Road, Leicester, LE5 4PW UK; Institute of Mental Health, Jubilee Campus, Room D16, Wollaton Road, Nottingham, NG8 1BB UK; Inclusion Healthcare, Charles Berry House, 45 East Bond Street, Leicester, LE1 4SX UK

**Keywords:** Fit note, Sickness certification, Statement of fitness for work, Recommendations, Achievability, Implementation

## Abstract

**Background:**

Although the UK fit note has been broadly welcomed as a tool to facilitate return to work, difficulties and uncertainties have resulted in wide variation in its use. Agreement on what constitutes the ‘ideal’ fit note from the perspective of all stakeholders is needed to inform best practice. A recent Delphi study conducted by the authors reached consensus on 67 recommendations for best practice in fit note use for employed patients. However, such recommendations are not necessarily followed in practice. The purpose of this study was therefore to investigate the perceived achievability of implementing these Delphi recommendations with a further reference panel of stakeholders.

**Methods:**

Potential participants were identified by the research team and study steering group. These included representatives of employers, government departments, trades unions, patient organisations, general and medical practitioners and occupational health organisations who were believed to have the knowledge and experience to comment on the recommendations. The consensus Delphi statements were presented to the participants on-line. Participants were invited to comment on whether the recommendations were achievable, and what might hinder or facilitate their use in practice. Free text comments were combined with comments made in the Delphi study that referred to issues of feasibility or practicality. These were synthesised and analysed thematically.

**Results:**

Twelve individuals representing a range of stakeholder groups participated. Many of the recommendations were considered achievable, such as improved format and use of the electronic fit note, completion of all fields, better application and revision of guidance and education in fit note use. However a number of obstacles to implementation were identified. These included: legislation governing the fit note and GP contracts; the costs and complexity of IT systems and software; the limitations of the GP consultation; unclear roles and responsibilities for the funding and delivery of education, guidance and training for all stakeholders, and the evaluation of practice.

**Conclusions:**

This study demonstrated that although many recommendations for the ideal fit note are considered achievable, there are considerable financial, legal, organisational and professional obstacles to be overcome in order for the recommendations to be implemented successfully.

## Background

In the UK, the GP is the main source of work support and advice and sickness certification. In 2010 the UK government replaced the sickness certificate with the Statement of Fitness for Work, or ‘fit note’ [1, 2] to reduce avoidable sickness absence and work disability. Previously, GPs could advise either that a patient should refrain from work, or need not refrain from work. The fit note now allows GPs to select either a ‘not fit’ or a ‘may be fit’ option in order to encourage GPs to focus, and advise, on what a patient may be able to do at work in order to facilitate a timely return, for example through adjusted duties or hours. However, although research studies have demonstrated that GPs and employers support the principle of the fit note, it is not being used as intended in practice. The ‘may be fit’ option is used infrequently [3], with employers reporting that the advice given is often limited, or unhelpful [4–6]. GPs, on the other hand, report that employers fail to provide the modifications required to enable patients to stay at work [7]. Patients report that GPs do not necessarily initiate discussions about work, even when issuing a fit note [8]. Qualitative research has highlighted the problems experienced by employers due to the poor legibility of handwritten fit notes [4, 6], and Dame Carol Black [1] recommended that electronic fit notes be introduced in the UK to improve communication between GPs and employers. The electronic fit note should have been rolled out to all GP practices by early 2013 [9] however recent studies indicate that the majority of fit notes are still hand-written [5, 10]. Of all the stakeholders (e.g., GPs, employers, employees), only GPs have been offered formal training related to fit notes; however although an optional half-day National Education Programme for GPs on managing the health and work consultation was positively received by participants [11], uptake was reportedly less than anticipated [5]. This is an area of growing international interest and importance – for example in March 2015 a new certificate of capacity was introduced in Australia, building on the fit note, focusing on what an injured patient can do as opposed to what they cannot [12].

Until recently, there has been no research on what constitutes an ‘ideal’ fit note. As a range of stakeholders are involved in the management of the fit note, recommendations that reflect the interests of all parties are more likely to be implemented into practice. Thus, in 2014 a modified Delphi study was conducted with the purpose of identifying how fit notes can best be used to aid return-to-work. A series of statements concerning best practice in fit note use were generated, based on previous data collected and published literature and presented to a panel of seventeen individuals identified as having expertise, standing, and/or knowledge in this area. The Delphi panel included general practitioners, employers, representatives of patient and employee organisations, occupational health practitioners, allied health professionals delivering work rehabilitation, and academics. The study has been reported in detail elsewhere [10], but in summary consensus (defined as ≥ 75 %) was reached for 67 statements, shown in Table 1. Key recommendations included increased use of electronic fit notes, changes to the fit note format and content, facilitating communication between stakeholders, completion of fit notes by other healthcare professionals, mandatory training in fit note use, auditing of fit notes, and the revision of existing guidance. These consensus statements have the potential to improve the effectiveness of the fit note in returning employees to work, however, recommendations do not necessarily change practice [13, 14]. In addition, free-text comments made by the Delphi panellists suggested that further investigation was needed to explore how easily the recommendations might be implemented; this was the aim of this study.

**Table 1:** Delphi statements where consensus was achieved

	Statement	% of respondents who agreed	Number of respondents
	General format and application		
1	Electronic/computer-generated fit notes should be standardised.	88.2 %	17
2	Electronic/computer-generated fit notes must exactly match the hard copy fit notes.	94.1 %	17
3	Fit notes should include a section stating whether or not the patient is employed.	82.3 %	17
4	The electronic/computer-generated fit note should have drop-down prompts for the GP, giving examples of the information they are expected to provide.	100 %	17
5	The comments section should be separate from the work modification tick boxes to encourage GPs to comment on 'not fit' notes as well as 'may be fit' notes.	76.4 %	17
6	GPs should be able to access a second (independent) opinion of a patient’s fitness to work.	100 %	17
7	The DWP should actively promote the use of electronic/computer-generated fit notes.	100 %	17
8	The DWP should actively monitor the use of fit notes.	88.2 %	15
9	Completion of all fields of the electronic/computer-generated fit note should be mandatory.	82.3 %	17
10	Electronic/ computer-generated notes must require the GP to select either the 'may be fit' or ‘not fit’ option.	82.3 %	17
11	GPs should have the option of selecting both fitness to work options ('not fit' and 'may be fit') if they qualify these choices with clear dates, duration and advice.	76.4 %	17
12	It is for the employer in conjunction with the employee, to consider and act on - or reject - the advice that they receive.	76.4 %	17
13	Other healthcare professionals with relevant training and competency should be able to complete fit notes.	88.2 %	17
14	If a patient has another job with different demands, the GP should complete the fit note to cover each job.	76.4 %	17
15	GPs need to understand the details of their patient’s work tasks in order to comment on the ‘functional effects’ of the patient’s condition.	82.3 %	17
16	Fit notes should include a section stating whether or not the patient is employed/ self-employed/ unemployed	75 %	16
17	The review section should be amended to a default statement ‘I will not need to assess your fitness for work again at the end of this period’ with the option to amend this if required.	75 %	16
18	There should be local audits of fit notes to ensure that fit notes are completed according to most up-to-date DWP guidelines.	82.5 %	16
19	Other healthcare professionals with relevant training and competency, who complete fit notes, should be Any Qualified Providers who have clinical data sharing set-up locally with GP systems.	75 %	16
20	GPs should provide as much information about the health condition on the fit note, relevant to their return to work, as the patient will consent to.	93.8 %	16
	Completion of the fit note		
21	Fit notes should be completed electronically.	82.3 %	17
22	Each section of the fit note must be completed.	82.3 %	17
23	The content of each section of the fit note must be discussed and completed with the patient’s knowledge and agreement.	88.2 %	17
24	If a patient has more than one condition affecting their ability to work, information and advice on the fit note should clearly distinguish to which condition this refers.	82.3 %	17
25	If medical terminology is used on a fit note, then a lay person’s version should also be provided e.g., CVA (stroke).	94.1 %	17
26	If a patient’s health condition is work-related (partially or fully) the GP should specify this on the fit note, with the patient’s consent.	76.5 %	17
27	If a patient has had, or is undergoing surgery, and with the patient’s consent, the GP should use the fit note to advise on expected post-operative complications and restrictions.	88.2 %	17
28	GPs should clarify the duration of recommended modifications on a fit note where possible.	94.1 %	17
29	GPs must complete the comments section of the fit note on both ‘not fit’ and ‘may be fit’ notes.	76.5 %	17
30	GPs must ensure there is no ambiguity as to the return to work date on the fit note.	76.5 %	17
31	If a patient’s symptoms are aggravated by work, then the GP should specify this on the fit note, with the patient’s consent.	88.2 %	17
32	With the patent’s consent, information on planned tests and treatment interventions impacting on the patient’s ability to work should be included on the fit note, with timescales where known.	82.3 %	17
33	Where possible, and with the patient consent, GPs should include information on the fit note as to the likely duration of reduced work capacity.	88.2 %	17
34	GPs should avoid using non-specific advice on work adjustments on a fit note e.g., light duties.	88.2 %	17
35	GPs should have the most up-to-date DWP fit note guidance available on their website and/or at their surgery.	88.2 %	17
36	When completing a fit note, the option of ‘may be fit’ should always be considered initially.	75 %	16
	Management of the fit note		
37	GPs must ask all employed patients whether there is anything about their health condition that makes it difficult to work, and if so, what this is.	82.4 %	17
38	GPs should state how the patient’s condition affects their ability to work (i.e., the functional effects of the condition) on both ‘not fit’ and ‘may be fit’ notes.	82.4 %	17
39	GPs must conform to the most recent fit note guidance published by the DWP.	82.4 %	17
40	It should be possible for patients to access the same GP for ongoing fit note consultations	76.5 %	17
41	GPs should ask the patient the extent of their employer’s occupational health provision and involvement when completing the fit note.	88.2 %	17
42	The option should be available, with patient consent, for fit notes to be emailed to the employer.	94.1 %	17
43	Employers must conform to the most recent DWP fit note guidance.	88.2 %	17
44	Organisations must have a timely mechanism for dealing with ‘may be fit’ notes.	94.1 %	17
45	Employers should ensure that sickness absence monitoring schemes do not discourage employees from returning to work before the expiry of their fit note, if they feel able.	94.1 %	17
46	There should be a defined period within which GPs complete reports requested by an employer or the employer’s occupational health provider.	88.2 %	17
47	Patients should not be discouraged from consulting their GP about a health problem that impacts on their ability to work during self-certificated sickness absence.	94.1 %	17
48	GPs should be able to write a fit note during the self-certification period, free of charge.	82.3 %	17
49	The use of email to send fit notes to patients’ employers should be piloted before a final decision is made.	88.2 %	17
50	The DWP should provide more detailed guidance to employers on best practice in the management of the fit note through their organisation.	94.1 %	17
51	Employers should have the most up-to-date DWP guidance available for employees on their website and/or at the workplace.	76.4 %	17
52	Reports requested from the GP by an employer or the employer’s occupational health provider should be completed within two weeks of the request being made.	88.2 %	17
53	Where reports are requested from the GP by an employer or the employer’s occupational health provider, there should be a standard fee.	82.3 %	17
54	Patients who seek consultation with their GP about a health problem affecting their ability to work should be able to request a face-to-face consultation.	87.5 %	16
55	Employees should contact their employer to discuss a 'may be fit' note within two working days of being issued with one.	87.5 %	16
	Communication about the fit note		
56	Fit notes should include GP contact details (phone, email) to facilitate discussion of the patient’s return to work should the employer wish to do so, and with the employee’s consent.	94.1 %	17
57	Employers should contact the GP by phone or email, with employee consent, if they have questions about the employee’s fit note.	88.2 %	17
58	Employers must ensure strict confidentiality in their management of fit note information.	100 %	17
59	Where necessary, and with patient consent, GPs should communicate with their patient’s employer to seek more information on the employee’s job and possible modifications.	82.4 %	17
60	Patients should be the primary channel of information between their GP and employer concerning the fit note.	88.2 %	17
61	Records of any contacts made between the employer and the employee’s GP should be sent to the employee (with the employee’s consent).	88.2 %	17
62	Employees should be consulted as to which members of staff within their organization will see the content of their fit note.	82.3 %	17
	Training		
63	GP fit note training should be incorporated into official GP training events (e.g., Practice Learning Time).	100 %	17
64	GP fit note training should be mandatory.	88.2 %	17
65	Employers must inform their workforce about how their organisation manages the fit note.	88.2 %	17
66	Employers must inform individuals as to any impact that work modifications advised on a fit note might have on their pay.	94.1 %	17
67	Training in the use of the fit note should include GPs, employers and patient/employee representatives so that each can hear the others’ viewpoint.	100 %	17

## Method

A list of potential participants was generated by the research team and study steering group. This list included key individuals known to have practical and/or theoretical knowledge of the fit note. In addition, organisations representing the main stakeholder groups were approached and were invited to identify individuals within their organisation who had the relevant knowledge and/or experience to participate in the study. Potential participants included representatives of employer organisations, government departments, trades unions, patient organisations, general and medical practitioners and occupational health organisations at both national and local level. Participants from the previous but related Delphi study [10] were also invited to participate in this study. All those identified were approached by email which included an information sheet about the study, and invited to respond.

Those who agreed to participate were each emailed an Excel file containing 67 consensus Delphi statements under five section headings, as shown in Table 1. A comment box was provided next to each statement/section and participants were invited to comment on whether the recommendations were i) achievable, and ii) what might hinder or facilitate their use in practice. Participants were given the option of either commenting on each section, or separately for each recommendation. Participants were assured that their name and/or the organisation they were employed by would not be identified in the study reports.

Free text comments from this study along with the free text comments pertaining specifically to issues of feasibility or practicality from the Delphi study [10] were transferred verbatim to a Word document and analysed thematically [15]. The comments were coded by the lead author (CC) who through a process of constant comparison identified significant or recurring words, phrases or concepts. The lead author then searched for and identified categories and overarching themes that best reflected the meaning of the data collected. These themes were then reviewed and agreed with members of the research team [FN, IP].

Ethical approval was obtained from the University of Nottingham Medical School Ethics Committee. Consent was assumed through participation.

## Results

Of the fifteen potential participants approached, one declined, five did not respond and nine agreed to participate. A further three panellists from the Delphi study also agreed to participate in this study. Thus a total of twelve participants were included. Comments referred to the achievability of the proposed recommendations, respondents’ personal opinions and organisational views. Six themes were identified: changing the format and method of completing the fit note; completion of the fit note; the fit note consultation process; workplace management of the fit note; communication between GPs and employers about patients’ fitness to work; promoting and supporting best practice in fitness use. As it has not been possible to report on the comments for every recommendation, those where there was minimal disagreement or comment on implementation issues have not been referred to specifically.

### Changing the format and the method of completing the fit note

-It was generally agreed achievable for the fit note to be completed electronically and for a standard template to be used. Implementation of these recommendations could be facilitated by agreement/co-operation between IT suppliers and the expiry of the supply of paper copies of the fit note. Implementation would be hindered by the unavailability, unreliability or unsuitability of software systems, in circumstances such as home visits where paper copies might still be required or if fit notes were to be completed by those who unable to use the system e.g.,

*‘not always possible –* e.g., *locums sometimes don’t have authorisation on the system to do them’*

Making changes to the format of the fit note, such as including the patient’s employment status and drop-down prompts for GPs, was considered achievable by most, but the required changes in IT specification could be a hindrance due to the *‘cost to the state to get GP software companies to do this’*. Format alterations might also be hindered by changes required by law, as the fit note is a legislated document. These recommendations could be facilitated by revised fit note guidance published by the Department for Work and Pensions (DWP), and the option of the GP entering ‘not applicable’ on a fit note (e.g., to accommodate matters of patient consent/confidentiality). Prompts should be clear, comprehensive and carefully selected – *‘if simple and limited then yes’* and should not replace the free text option or deter individual tailoring of fit notes. Completion could be hindered by increasing the number or complexity of sections included in the fit note and thus the length of the consultation. Most considered it achievable for all fit note fields to be completed but there were differences of opinion as to whether it was achievable that both ‘not fit’ and ‘may be fit’ options could be selected on the same fit note (to accommodate both a period of absence followed by a period of modified work), and for fit note advice to distinguish which condition is referred to where a patient has more than one health condition.

There were differences of opinion as to whether it would be achievable for other healthcare professionals with relevant training and competency to complete fit notes - *‘achievable and necessary given the number of consultations being delegated to nurses, physiotherapists and non-medical mental health practitioners’.* This recommendation could be facilitated by healthcare professionals having access to patient records to gain a full picture of the individual’s health. Currently there is limited sharing of patient data/notes and there are issues of confidentiality and access to case notes of different professions and providers. Implementation of the recommendation could also be facilitated by clarity as to which categories of staff (e.g., allied health professionals, nurses, occupational health practitioners) would be able to complete fit notes, and whether these staff would also have the other responsibilities of the GP regarding fit note completion. It could be hindered by the fact that it would require a change in the law, and how the delivery, content and assessment of training was monitored – *‘I suspect it will require a lot of new regulations and training for other professions’.* It could be facilitated by the introduction of the new ‘Fit for Work Service’ - a state-funded occupational health assessment and advisory service [16, 17] - but hindered by a lack of suitably qualified professionals. There was also a view expressed that patient consent should be obtained if a fit note were to be completed by another healthcare professional. There were differences of opinion as to whether it was achievable that these individuals should be a designated Any Qualified Provider. (Any Qualified Provider is a means of commissioning certain NHS services in England under which any provider who is able to provide a specific service and meets the required minimum standards can be listed as a possible provider).

### Completion of the fit note

There was overall agreement that it should be achievable for patients to be fully *aware* of what the GP has written on the fit note and the reasons for completion. However there were differences of opinion as to whether patient *agreement* would be achievable. Some participants considered that patient agreement with fit note content was not only achievable but should already be routine practice. Others felt that gaining patient agreement could limit objectivity, or would lengthen the consultation, and should only be carried out ‘where relevant’. Implementation of the recommendation could be hindered by telephone consultations ‘*achievable if all appointments are face-to-face; from experience there seems to be an increasing number of telephone consultations – the patient will not see the content of the fit note until they collect it and it will be harder for them to comment on or disagree on content’*.

There were differences of opinion as to whether it was achievable for the GP to provide as much information about the health condition, relevant to their return to work, as the patient would consent to. There was a view that the main focus of the fit note is considered to be on function and advice rather than providing details on the diagnosis or condition; thus it should be seen as an option and not an expectation, although ‘*the fit note details will be used by an employer and the more information provided the better the employer will be able to assist the employee with their return to work’*. Implementation of this recommendation could be facilitated by GP training on conducting functional assessments, and hindered by limited consultation time.

Most responders considered it achievable that if medical terminology is used on a fit note then a lay version should also be provided although some believed that this was unrealistic or should be at the GPs’ discretion. Implementation of this recommendation could be facilitated by its inclusion in the DWP guidance documentation.

There were differing opinions as to whether it was achievable that GPs should understand the details of their patients’ work tasks in order to comment on the ‘functional effects’ of the patient’s condition:

*‘Yes, GPs can comment on the function in general terms without understanding the job’*

And

*‘Without detailed knowledge of the type of work being done, this may be difficult to do’*

Implementation of this recommendation could be facilitated by better GP training, by encouraging GPs to ask patients about their work tasks, and by the provision of a generic functional task list as a prompt. However, implementation could be hindered by a number of factors including lack of time in the consultation, the reliability of the patient’s report, consultations where English is not the first language, the complexity of the patient’s job, and by the perception that GPs need occupational health training to comment on the functional effects of the patient’s condition. It was also reported that this recommendation was actually contrary to current DWP guidance, so this would need to be revised.

Most respondents believed it to be achievable that the ‘may be fit’ option should always be considered initially when a GP is completing a fit note: *‘the default should be ‘may be fit’*. This recommendation could be facilitated by its inclusion in the DWP guidance notes and by an accurate interpretation of the term ‘may be fit’. Implementation could be hindered by concerns about this consideration potentially increasing the GP workload, for example by increasing the length of the consultation. Some participants believed there to be circumstances where such a consideration would be inappropriate, e.g., in ‘short term’ illnesses, or following major surgery. There was a difference of opinion however as to whether it was achievable for GPs to clarify the duration of recommended modifications on a ‘may be fit’ note.

It was generally considered achievable that if a patient’s health condition is work-related (partially or fully) or aggravated by their work for the GP to specify this on the fit note, with the patient’s consent. Implementation of this recommendation could be facilitated by the use of a tick box and by its inclusion in DWP guidance documents. There were however views expressed that judging a condition as work-related/aggravated is subjective and that the GP may not be best placed to judge this. There were also concerns about whether this recommendation might lead to a significant increase in the reporting of work-related conditions, especially for mental health issues. Reasons for this were not elaborated on, but are perhaps associated with a culture shift away from the ‘work stress’ movement, which is based on the premise that work can make a person mentally unwell [18] - *‘I am not sure the GP is always best placed to say a condition is work-related as work is normally the first area to be blamed, when in reality it is often a combination of factors’.*

Most participants considered it achievable that the fit note should advise on postoperative *restrictions*, although not necessarily *complications*, and that this recommendation would be facilitated by hospital departments issuing appropriate fit notes. There were differences of option as to whether it was achievable for GPs to provide information on tests and interventions that may impact on the patient’s ability to work as this would increase the time and complexity of the consultation and require knowledge of the patient’s work.

There was a difference of opinion as to whether GPs should avoid using non-specific advice on work adjustments on a fit note, e.g., ‘light duties’. Implementation of this recommendation could be facilitated by better GP training and the GPs’ understanding of the patient’s work, and longer consultations – *‘it may slow down the consultation but very non-specific advice will not help the employer or the employee’.* There was also a view expressed that it is not the role of the GP to offer specific advice as this is the role of occupational health.

There were also differing views as to whether it would be achievable to recommend that GPs must complete the comment section on both ‘not fit’ and ‘may be fit’ notes. It was thought that implementation could be facilitated by better GP training, and revising the DWP guidance. It could be hindered by potentially lengthening and complicating the consultation, and by patients not wishing to disclose too much information in the initial period of absence.

### The fit note consultation process

There were differences of opinion as to whether it would be achievable for GPs to ask all employed patients whether there is anything about their health condition that makes it difficult to work – *‘unfortunately I don’t think it often features as part of GP history taking’* and ‘*again major workload implications’.* There were concerns that this might result in more patients being ‘signed off’ and should only be considered where ‘relevant’. Others considered that this was part of routine history-taking, and that the same should be asked of unemployed patients. Achievability could be facilitated by better GP training. Implementation could be hindered by patients who are unwilling to disclose, or who perceive that they have a condition which affects their ability to work but in reality does not.

It was seen as achievable by most respondents that patients who seek a consultation with a GP about a health problem affecting their work should have the option of a face-to-face or telephone consultation, and that this is included in the current DWP guidance. However, the recommendation would be hindered by the fact that not all GPs do offer telephone consultations, and by resource issues, as telephone consultations are less time consuming. Implementation could also be hindered by those who considered it to be the GP who decides which method is appropriate and not the patient. There was also a view that face-to-face consultations improved the ‘patient experience’.

There were differences of opinion as to whether it was achievable that patients should be able to access the same GP for ongoing fit note consultations. Implementation of this recommendation would be hindered by the set-up and resources of GP practices, annual leave, sick leave and GPs’ other work commitments, and there were concerns that it could lead to a delay in the issue of a fit note. The recommendation could be facilitated by better training of GP receptionists. There was a view that electronic record keeping and a ‘properly completed note’ would make it less critical that the patient sees the same GP.

It was generally considered achievable that patients should not be discouraged from consulting their GP about a health problem that impacts on their ability to work during self-certificated sickness absence, and that GPs should issue fit notes free of charge within this period. Implementation of this recommendation would be facilitated by raising patient awareness, situations where the duration of the fit note was likely to be longer than the self-certification period, and if the purpose was to provide additional information to assist the employer and employee. It would be hindered by lack of available GP appointments, the current GP contract – (GPs have discretion at the moment) and time – *‘we have discouraged patients for years about not needing to attend during this time and that if advertised it would have a negative impact on access for other patients’*. Some considered that this recommendation should depend on the health conditions - for example ‘GPs do not necessarily want to see patients with upper respiratory tract infections’ – and that need should primarily be determined by clinical need.

There were differences of opinion as to whether it would be achievable for fit notes to be emailed to the employer, with patient consent. Implementation of this recommendation would be hindered by the fact that it is not in the current GP IT system contract and by the costs such a change would entail. There were concerns about issues of consent, confidentiality, accuracy of email addresses, adding to the GP workload, and whether this method would take away the responsibility from the patient. There was however a view that it could enable more direct and timely communication in some circumstances where employees are less proactive, or where there are obstacles to communication such as language barriers. It was agreed that this method should be piloted before any decisions were made.

### Workplace management of the fit note

The majority of responders thought it achievable for employers to ensure strict confidentiality in the management of fit note information. Implementation of the recommendation would be facilitated by employees knowing about policies concerning the confidential management and distribution of fit notes before the need arises.

There were differing opinions as to whether employees should be *consulted* as to who sees the content of their fit note – some considered that employees should instead be *informed*. It was perceived to be the responsibility of organisations to address this in their sickness absence policies and procedures. Employees ‘*should be assured that fit notes are held securely, and who has access’*.

Most respondents considered it achievable for organisations to have a timely mechanism for dealing with ‘may be fit’ notes and for employees to contact their employer to discuss a ‘may be fit’ note within two working days of being issued with one. This would be facilitated by organisational sickness absence policies reflecting the process and timescales for accommodating adjustments. There was a view that this may not be possible depending on the specific industry and days worked by the patient, and should not therefore be mandatory.

Most respondents considered it achievable for employers to ensure that sickness absence monitoring schemes do not discourage employees from returning to work before the expiry of their fit note, if they are able. Implementation of this recommendation would be facilitated by the organisation addressing this issue in their sickness absence policies and procedures and that ‘*much more work is required on educating that the note is advisory – if the employee feels well enough and follows work procedures to report an earlier return to work then this is acceptable’.*

### Communication between GPs and employers about patients’ fitness to work

There were differences of opinion as to whether it would be achievable for GP contact details (phone, email) to be included on the fit note to facilitate discussion of the patient’s return to work, and for employers to contact the GP. This could be hindered by perceived obstacles of patient consent *– ‘it is difficult to be certain that the employee has given consent in a phone or email exchange’*, GP time/workload and GP role. There were concerns about potential confidentiality breaches and that, according to the DWP guidelines, the GP is not required to issue job-specific advice. Such communication could also incur a cost to the employer as GPs could charge for the provision of additional information under the terms of their contract.

GPs are under no obligation to check emails daily and may not see a reply from an employer, therefore the use of these methods should not be expected as routine, although may be useful in some specific cases. If contact were to be made, accurate records with copies sent to the patient would be seen to aid trust. The keeping and provision of records is achievable, but may be a resource issue for both employer and GP.

There were also differences of opinion as to whether it would be achievable for GP reports, requested by an employer or the employer’s occupational health adviser, to be completed within two weeks of the request being made, and if there should be a standard fee. There were views expressed that a) timelines are helpful to guide the GP, and that this is good practice, but compliance would be hindered by ‘*heavy workload demands and more important clinical priorities’* and b) that GPs do not necessarily have the ability to produce useful reports. There were additional views that a) not all employers will pay b) employers might withhold payment for inadequate reports c) requests and reports can vary in complexity. The quality of reports might be facilitated by providing GPs with more guidance as to the content of reports, or standard proformas. Implementation of the recommendation would require further legislation to revise the GP contract.

### Promoting and supporting best practice in fit note use

Most respondents thought that GPs should conform to current DWP fit note guidance in fit note use and that fit note training should be incorporated into official training events. Implementation of this recommendation could be facilitated by sufficient resources and the support of those bodies responsible for GP education and local GP Clinical Commissioning Groups. Uptake of training and use of guidance could be facilitated by: audits of existing training, for example by the Royal College of General Practitioners or the Care Quality Commission; by on-line modules; by addressing training needs *‘need better GP training – currently no penalty for the GP not doing this’.* However, these recommendations could be hindered by the view that it is already part of Good Medical Practice that GPs stay ‘up to date’, and that training is primarily an issue for ‘new starters’. The issue was also raised that guidance, as such, cannot be mandatory.

Most respondents thought it was achievable that organisations should conform to DWP guidance and for organisations to inform their workforce as to how they manage the fit note, however mandating this recommendation was again considered unrealistic – *‘as guidance it is not practical to specify that it must be conformed to’.* Most also thought it achievable for the DWP to provide more detailed guidance to employers on best practice in the management of the fit note through their organisation, although there was a view that the DWP may not be best placed to give this guidance. Implementation would be facilitated by better training of employers, more detailed fit note guidance and the resources required to produce it.

It was generally perceived to be achievable for GPs to have the most up-to-date DWP fit note guidance available on the practice website and/or at the practice and available for patients to see. Implementation of the recommendation would be hindered by those who perceive that this information should be accessed through the DWP website, and that dissemination is the role of the DWP rather than the GP.

The majority of respondents also believed it to be achievable for employers to have the most up-to-date DWP fit note guidance available on their website and/or at the workplace. Implementation would be hindered by those who perceive that this information should be accessed through the DWP website, and that the DWP material may not be suitable.

Most respondents believed that other key stakeholders should be included in fit note training, and that training should include occupational health professionals and hospital doctors, but that it may not be practical - ‘*in an ideal world would be nice – but probably excessive and unachievable*.’

With regard to promoting and monitoring the use of the electronic fit note by the DWP, most believed this was achievable, that the use of electronic fit notes should be audited and published, and for local fit note audits to be conducted. Implementation of this recommendation could be facilitated where audits were not punitive or increased GP workload, and by the DWP setting a date by which all fit notes should be computer-generated. Monitoring could be facilitated by the audits being conducted by the Care Quality Commission and/or the Royal College of General Practitioners. Implementation would be hindered by those who perceived audits to be intrusive, or that their usefulness would be limited if restricted to reporting only on quantitative data rather than free-text fit note comments.

## Discussion

The results of this study demonstrated that many of recommendations on best practice fit note use were considered achievable by the majority of participants, particularly the use of standardised electronic fit notes, the completion of all sections, the inclusion of drop-down prompts and recording patients’ employment status. It was also thought feasible for employers to ensure confidentiality in the management of fit note information and timely mechanisms for dealing with ‘may be fit’ notes, that employers and GPs should conform to the most up-to-date DWP guidance, and for the increased availability and uptake of training. However, comments made by participants identified a number of key issues and concerns that may impact on implementation of the recommendations. They also demonstrate the diversity of opinions about how the fit note should best be used and some of the underlying reasons why it has not yet reached its potential, for example due to varying interpretations of how the fit note should be completed.

The implementation of several of the recommendations would rely on GP practice resources. Available and reliable IT systems and software are required to effectively manage the electronic fit note. GP practices in the UK have different IT providers and local operating systems, and different implementation timetables for the fit note [9]. The electronic version is thought to facilitate legible, comprehensive and effective fit notes [19]. However, although a standard design and output should be achievable, it seems that practice resources, inconsistencies and compatibility problems in IT systems may be impeding implementation of the electronic fit note. Changes in IT specification could be difficult and costly to carry out and it is unclear how these would be funded. Providing greater continuity of consultations, and for any potential increase in face-to-face consultations, including those within the first seven days of absence, would also incur costs. Greater priority needs to be given to the fit note, but this seems unlikely when GPs report a decline in practice income alongside increasing costs of running a practice [20].

Several recommendations would require legislation in order to be implemented. Any alterations to the fit note format, for example, adding a section on employment status, or separating the free text comment box from the work modification options would require a change in the law. The completion of fit notes by other healthcare professionals would also require legislative change, as would any amendments to the GP contract such as expediting medical reports requested by employers and increasing the 10 min consultation time. The current GP contract dates from 2004 [21], although amendments are made to the regulations on an annual basis.

The study findings highlight discrepancies in the perceptions and expectations of the GP role in fit note completion and issues of health and work generally that have been reported elsewhere. Lack of clarity about the extent that GPs need to know about their patients’ work, and the content and detail of the advice they are expected to provide would seem to present key obstacles to the implementation of recommendations. Different perceptions as to the meaning of the term ‘functional effect’ and the extent to which this relates to work, underline the need for training and improved guidance. For example, although current DWP guidance states that GPs do not need to understand the detail of their patients’ jobs, they are however expected to be able to suggest how the patients’ jobs – or indeed ‘any work’ - might be modified. The decision not to include a definition of this phrase in fit note rules was based on the reason that it would be too complex [22], however the findings of this study would suggest that a definition is needed. The lack of information provided by GPs on patients’ functional ability has been reported elsewhere. Norwegian GPs have reported on their difficulty in assessing and communicating on function [23, 24], and in a study of Swedish sickness certificates, only one-third of GPs reported on patients’ activity limitations despite the introduction of new guidelines and implementation strategies [14, 25]. Studies set within workers’ compensation schemes also report on discrepancies between the lack of GPs’ knowledge about their patients’ work and the functional advice they are expected to provide [26, 27].

It was suggested in this study that some of the recommendations considered to be achievable might be facilitated by their inclusion in the DWP guidance documents. Employers would also need to take a more active role in promoting good practice in fit note management through their sickness absence policies and procedures. The current DWP guidance for employers does not address key aspects such as managing confidentiality, the effect of absence policies on return-to-work, or how quickly employers should attend to ‘may be fit’ notes. Further revision of the guidance is indicated, however, unless such guidance is disseminated more widely and effectively, this may be of little benefit. Other studies have reported that GPs, employers and employees are not necessarily aware of the DWP guidance, or its revisions [10, 28]. It is unclear whose role and responsibility it is to disseminate guidance – to employees as well as employers and GPs - and who should fund this. Making guidance available on websites is useful, but it is not well-accessed [28, 29].

It was also suggested that further training of GPs and employers would facilitate the implementation of several of the recommendations, but again there are issues of roles, responsibilities and resources to be addressed, particularly if training is to be made mandatory. It would appear that the provision of joint training where GPs, employers (including occupational health) and employees might learn from each other’s experiences and perspectives, whilst attractive in theory, has considerable practical issues to overcome. Evaluating the impact of training on the completion and management of fit notes could be measured through local audits, but again, the costs and responsibilities need to be allocated.

A recurring theme from the comments made in this study referred to concerns about the recommendations increasing GPs’ workload. Other studies have reported that the assessment of patients’ work ability is not perceived to be a priority for GPs, and that the fit note is not considered to be a key part of their workload. It would appear vital therefore that training in the management of the fit note consultation emphasises the importance of the GP role and how GPs can make best use of the time available to them.

This study highlights the difficulties of implementing change in General Practice. Starfield et al. [30] have argued that disseminating practice in this area remains a ‘worldwide challenge’ while Gilbert et al. [31] feel that the high level of professional autonomy within primary care impedes the achievement of large-scale change. Across all healthcare professions, the difficulties of translating high quality evidence into routine practice are widely reported [32–34]. Studies have demonstrated that the effective adoption of evidence-based practice is influenced by a number of factors concerning the nature of the practice, the practitioners adopting it, and the environment that they work in [35, 36].

Social influence is one factor that plays an important part in the embedding of new practices into everyday work [36, 37]. Within this, the role of opinion leaders, change agents and social norms have been identified as critical ingredients in making changes in practice [38]. Identifying the appropriate opinion leaders and change agents in relation to the adoption of the fit note, which currently involves a number of stakeholders outside the medical profession (e.g., DWP, and employers), may be an important step. Identifying and addressing local barriers is also important in order to successfully implement change in clinical practice in primary care. A systematic review of the effectiveness of healthcare interventions tailored to address identified barriers to change compared to interventions which were not tailored concluded that tailored interventions are more likely to improve professional practice [39]. It may be that further work implementing the Fit Note is required, and that this may be more effectively undertaken at a more local level, e.g., by Clinical Commissioning Groups where local barriers can be addressed and where local change agents and opinion leaders can be utilised more effectively. Further research and professional consultation is also required in primary care to focus on how maximum returns can be generated whilst maintaining a manageable workload for GPs, as this appears to be a key barrier to implementing ideal fit note practice.

There are some limitations to this study. First, there were no fixed criteria for recruitment, or means of validating the knowledge and experience of the participants. However, participants were either known to the study team or held key posts within the organisations they represented. Second, there was no independent coding of the comments, however, these and the themes were discussed and agreed between members of the research team. Thirdly, participation may have been limited by participants’ concerns about being identified, and might have been improved by extending the response period – the achievability exercise was conducted during the summer break. Finally, although consensus was reached for the Delphi statements, it is acknowledged that these are opinions and not facts.

This is the first study to examine the feasibility of implementing recommendations for the ‘ideal’ fit note. Although there were a small number of participants in both the Delphi study and the feasibility reference panel these individuals represented the main stakeholder groups. Their comments have highlighted important factors that will help, or hinder, implementation of the recommendations in practice. These findings should be used to inform improvements in fit note use, including revisions to the fit note itself, completion, management, guidance and training.

## Conclusions

This study has highlighted the considerable financial, legal, practical and professional challenges to the use of the fit note in practice. However it has also demonstrated the potential of such changes to improve fit note use and thus reduce avoidable sickness absence and work disability.

### Availability of supporting data

We have no budget to make this available formally but we are happy to share data with colleagues. We would make paper available on the university repository if permission was given for this.

## Abbreviations

DWP: Department for Work and Pensions
GP: General Practitioner
IT: Information Technology
UK: United Kingdom
